# Rapid and Flexible Platform To Assess Anti-SARS-CoV-2 Antibody Neutralization and Spike Protein-Specific Antivirals

**DOI:** 10.1128/mSphere.00571-21

**Published:** 2021-07-28

**Authors:** Debora Stelitano, Stuart P. Weisberg, Monica P. Goldklang, Yun Zhu, Francesca T. Bovier, Gavreel F. Kalantarov, Giuseppe Greco, Didier Decimo, Gianluigi Franci, Michele Cennamo, Giuseppe Portella, Massimiliano Galdiero, Cyrille Mathieu, Branka Horvat, Ilya N. Trakht, Anne Moscona, Michael A. Whitt, Matteo Porotto

**Affiliations:** a Department of Pediatrics, Columbia University Vagelos College of Physicians and Surgeons, New York, New York, USA; b Center for Host-Pathogen Interaction, Columbia University Vagelos College of Physicians and Surgeons, New York, New York, USA; c Department of Experimental Medicine, University of Campania “Luigi Vanvitelli,” Napoli, Italy; d Department of Pathology and Cell Biology, Columbia University Vagelos College of Physicians and Surgeons, New York, New York, USA; e Department of Medicine, Columbia University Vagelos College of Physicians and Surgeons, New York, New York, USA; f Department of Anesthesiology, Columbia University Vagelos College of Physicians and Surgeons, New York, New York, USA; g Beijing Pediatric Research Institute, Beijing Children's Hospital, Capital Medical University, Beijing, China; h CIRI, Centre International de Recherche en Infectiologie, Team Immunobiology of the Viral infections, Univ Lyon, Inserm, U1111, CNRS, UMR5308, Université Claude Bernard Lyon 1, Ecole Normale Supérieure de Lyon, Lyon, France; i Department of Medicine, Surgery, Dentistry, University of Salerno “Scuola Medica Salernitana,” Salerno, Italy; j Department of Translational Medical Sciences, University of Naples Federico II, Naples, Italy; k Department of Microbiology & Immunology, Columbia University Vagelos College of Physicians and Surgeons, New York, New York, USA; l Department of Physiology & Cellular Biophysics, Columbia University Vagelos College of Physicians and Surgeons, New York, New York, USA; m Department of Microbiology, Immunology and Biochemistry, Health Science Center, The University of Tennessee, Memphis, Tennessee, USA; University of Kentucky College of Medicine

**Keywords:** SARS-CoV-2, spike protein, immunity

## Abstract

The COVID-19 pandemic caused by severe acute respiratory syndrome coronavirus type 2 (SARS-CoV-2) is ongoing and has shown the community that flexible methods for rapidly identifying and screening candidate antivirals are needed. Assessing virus-neutralizing activity of human serum to monitor population immunity and response to infection and vaccination is key to pandemic control. We developed a virus neutralization platform strategy that relies only on bioinformatic and genetic information of the virus of interest. The platform uses viral envelope glycoprotein cDNAs to set up an assay that mimics multicycle infection but is safe and, therefore, amenable to biosafety level 2 (BSL2) conditions for viruses that require BSL3 facilities (e.g., SARS-CoV-1 and SARS-CoV-2). As a complement to this platform, we present a new cell-based immunofluorescent (CBI) assay that uses SARS-CoV-2 spike protein (S)-expressing cells to accurately measure the neutralization potential of human sera and is readily adaptable to variants of concern. These methods should be useful additions to the tools for assessing antiviral immunity, whether acquired via natural infection or vaccines.

**IMPORTANCE** Assays for rapid biosafety level 2 (BSL2) evaluation of neutralizing properties of antibodies acquired via natural infection or through vaccination is urgently needed. Here, we propose a combinatorial approach in which sera are screened for SARS-CoV-2 spike protein (S) binding using a cell-based immunofluorescent (CBI) assay, and positive samples are further evaluated in a pseudotyped viral multicycle infection-mimicking protocol under BSL2 conditions.

## INTRODUCTION

The recently emerged severe acute respiratory syndrome coronavirus 2 (SARS-CoV-2), the causative agent of coronavirus disease 2019 (COVID-19), has infected tens of millions of people. Vaccination is now underway globally, and long-lasting immune response will be the key to success in curtailing the pandemic. Assessing humoral immune response consistently and on a broad scale is important for recovering patients, as well for the vaccinated population. Methods for screening the neutralizing properties of human sera under biosafety level two (BSL2) conditions are valuable, since live SARS-CoV-2 requires scarce biosafety level three (BSL3) facilities.

**FIG 1:**
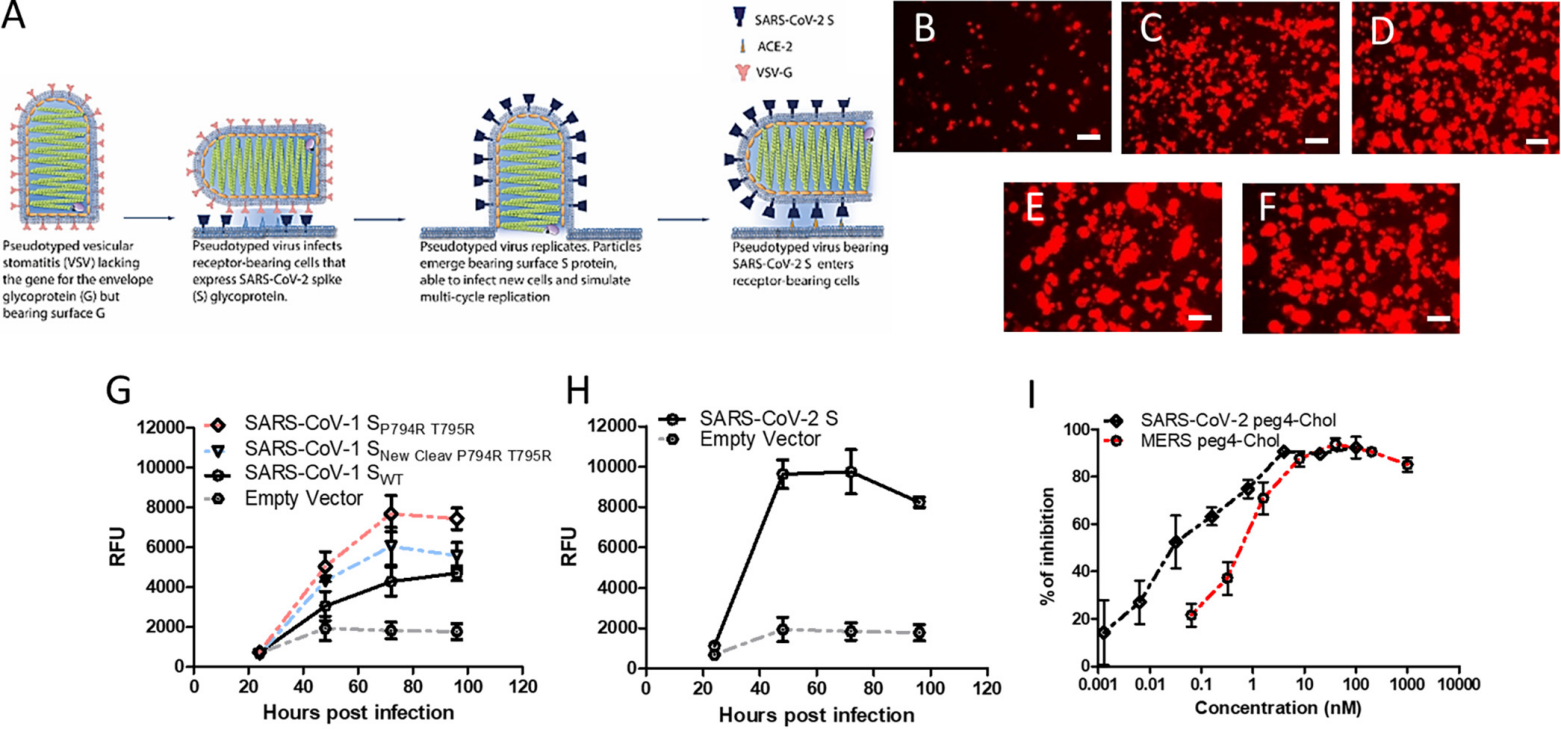
Adaptation of multicycle viral infection (MCI) assay to SARS-CoV-1and SARS-CoV-2. (A) VSV-RFP ΔG* virus pseudotyped with VSV-G infects HEK293T cells that express the SARS-CoV spike (S) protein and its receptor. Virus replicates in the cells and acquires the S protein upon budding from the host membrane. VSV-RFP ΔG* virus pseudotyped with S infects CoV receptor-bearing cells. Vero cell overlay increases the signal. (B to F) Cells were transiently transfected with either empty vector (B), SARS-CoV-1 S_WT_ (C), SARS-CoV-1 S_P794R T795R_ (D), SARS-CoV-1 S_New Cleav P794R T795R_ (E), or SARS-CoV-2 S (F), and then infected with VSV-RFP ΔG* virus pseudotyped with VSV-G. Relative RFP fluorescence intensities were measured at 24, 48, 72, and 96 h. (G and H) Data show the mean ± SEM of three independent experiments. (I) MERS and SARS lipopeptides inhibit SARS-CoV-2 S MCI. Cells coexpressing SARS-CoV2 S and ACE-2 receptor were infected as in panels B to F in the presence of the different peptide concentrations (*x* axis) at 48 h. Postinfection, the relative fluorescent units (RFU) were measured and used to calculate the % of inhibition compared to the control (untreated). See Materials and Methods for details. Data represent the mean ± SEM from three independent experiments.

Two main screening strategies have been deployed so far for SARS-CoV-2: lentiviral based pseudotyped virus systems (1–3) and vesicular stomatitis virus (VSV)-based systems with either classical pseudotyped VSV or recombinant virus encoding the SARS-CoV-2 spike (S) protein (4–7). The lentiviral and classical VSV pseudotyped systems use a single-infection S protein-pseudotyped virus stock. The recombinant VSV encoding the SARS-CoV-2 spike is a fully competent replicative virus. Distinct from this, we adapted our assay (8) to combine the safety of a replication-incompetent virus with the simplicity and robustness of a self-replicating virus. We previously developed several strategies to evaluate antiviral activity and neutralization properties against BSL4 and BSL3 pathogens, and adapted these methods to high-throughput screening (HTS) (8, 9). For SARS-CoV-2, we transfect cells with plasmids that encode SARS-CoV-2 S protein, then infect the cells with VSV that lacks the gene for the VSV entry glycoprotein G, but is pseudotyped with G. This is basically a miniaturized format of the procedure for generating a single-cycle VSV pseudotype (see also Fig. 1A). This methods permits a “multicycle” infection, since the pseudotyped virus enters using G but exits bearing S, then re-enters new cells using S. This is done with either SARS-CoV-1 or SARS-CoV-2 S and can be performed safely under BSL2 conditions, does not require generation of new pseudotyped viruses for each emerging S variant, and produces a qualitative assessment in 24 h and quantitative results within 48 h.

We first assessed SARS-CoV-1 and SARS-CoV-2 antiviral peptides and patient sera for their ability to inhibit multicycle infection. The assay is performed in 96-well plate format with a quantitative fluorescent readout. Ramp-up time is minimal, since the assay does not require specific pseudotyped viruses to be produced for the first step, i.e., the VSV-G pseudotype required for the first step can be prepared in advance. These features permit rapid screening of antiviral agents and antibodies in cells that express the relevant host factors (e.g., receptors and proteases [10]) and make the method adaptable to high throughput. As mentioned above, the system is readily modified for new dominant S variants (e.g., D614G) (11–13), and for newly emerging variants of concern (14–16). As a complement to the multicycle replication assay for assessing patient sera (which we recently validated for SARS-CoV-2 S [17]), we developed a cell-based immunofluorescent assay using S-expressing cells that rapidly measures neutralization activity of human sera under BSL2 conditions. Neutralization data in this cell-based immunofluorescent assay correlates directly with live virus neutralization activity.

## RESULTS

### Multicycle infection assay for SARS-CoV-1 and SARS-CoV-2 spike proteins under BSL2 conditions.

The betacoronavirus spike (S) protein virion mediates attachment, receptor binding, and membrane fusion. SARS-CoV-2 S uses the human angiotensin-converting enzyme 2 (hACE2) for entry (17) and requires cleavage by a host protease (10) to generate the subunits S_1_ and S_2_ in order to mediate viral entry. Traditional pseudotyped viruses bearing heterologous surface glycoproteins must be generated anew for each new emerging variant. This adds to the lead time for an assay for each new variant. Such pseudotyped virus entry assays rely on the readout of a single cycle reporter (18), or on viral evolution if the S is included in the VSV genome (4). The pseudotyped virus used in our system for initial infection uses VSV lacking the gene encoding G (“ΔG”) and pseudotyped with VSV G. These pseudotyped viruses can easily be produced at titers higher than most heterologous envelope protein-bearing pseudotyped viruses. The first entry event (at a very low multiplicity of infection [MOI]) is mediated by VSV G but does not permit subsequent rounds of infection unless the target cells are transfected with a viral envelope protein. By supplying the envelope glycoproteins of the “new” virus in *trans*, virus that is produced and released is a pseudotyped “new” virus (with the VSV envelope but altered tropism) that mimics native virus in terms of infection (Fig. 1A). Even though the initial infection event is mediated by VSV-G pseudotyped virus, the second and subsequent rounds will be mediated by SARS-CoV-1 and -2 S protein.

For SARS-CoV-1 and -2 assays, human HEK 293T cells in 96-well plates were transfected with empty vector (Fig. 1B) or plasmid encoding the S proteins of SARS-CoV-1 (Fig. 1C, E to G) and SARS-CoV-2 (Fig. 1F and H), along with SARS-CoV-1 and -2 specific receptor (hACE2) (10). Plasmids encoding green fluorescent protein (GFP) were transfected concomitantly to assess the efficiency of transfection. Transfected cells were then either infected with pseudotyped VSV-ΔG carrying red fluorescent protein (RFP, to permit visualization of infected cells by fluorescence microscopy and quantitation by spectroscopy) or remained uninfected (data not shown). Vero cells were overlaid after infection to increase the signal (without this step, RFP values were significantly lower; see the Materials and Methods). Control wells with cells transfected with an irrelevant plasmid were also infected with the pseudotyped virus (see empty plasmid curves in Fig. 1G and H). The virus underwent multicycle infection (MCI) in S-transfected cells, as indicated by an increase in RFP relative fluorescent units (RFU) over time after infection (Fig. 1G and H). For wild-type (wt) SARS-CoV-1 S, the dynamic range was lower, and maximal only after 96 h. To increase the dynamic range of the assay, we modified the SARS-CoV-1 S by introducing mutations at the S_1_/S_2_ cleavage site to increase infection readiness (see Materials and Methods). For wt SARS-CoV-2 S, the difference in read-out between the S-transfected target cells and the control-plasmid transfected target cells (Fig. 1E and B, respectively) was more than 4-fold, and differential RFU was measureable as early as 48 h posttransfection. No modification to the SARS-CoV-2 S was necessary to improve the dynamic range in this assay and it was recently used to detect neutralizing antibodies in clinical samples (19).

### Validation of the SARS-CoV-2 S MCI assay using fusion-inhibitory lipopeptides.

We validated the MCI assay using entry inhibitors that target the SARS-CoV-2 S protein. During the first steps of the SARS-CoV-2 viral entry process, S forms an extended intermediate and the fusion peptide portion of S is inserted into the target cell membrane. The extended intermediate then refolds via association of two heptad-repeat (HR) domains of the fusion subunit, one near the N terminus (HRN) and the other near the C terminus (HRC), into a six-helix bundle (6HB) assembly. This conformational rearrangement of S leads to cell membrane and viral envelope merger and viral entry (reviewed in references 20–22). In previous work, we generated lipid-conjugated peptides that target the HRN domain of MERS-CoV or SARS-CoV-2 and block the refolding step of S, thereby preventing viral entry, and assessed their effectiveness in fusion assays and against live virus (23, 24). MCI assays in the presence of the indicated concentrations of both MERS (23) and SARS HRC (24) lipopeptides were performed and, as anticipated, showed that both peptides potently inhibit SARS-CoV-2 MCI, with the SARS HRC lipopeptide superior to the MERS HRC lipopeptide (Fig. 1I). In Table S1 in the supplemental material, we show a direct comparison of the inhibitory concentration (IC) of the lipopeptides that inhibit the RFP signal by 50% (IC_50_) and 90% (IC_90_) in MCI assays compared to the IC_50_ and IC_90_ in plaque reduction assays with live virus (23, 24). The relative potency of the two lipopeptides is maintained, although the MCI signal is blocked at considerably lower concentrations than the live virus signal (Table S1).

### Cell-based immunofluorescent assay versus S correlates with MCI neutralization.

**FIG 2:**
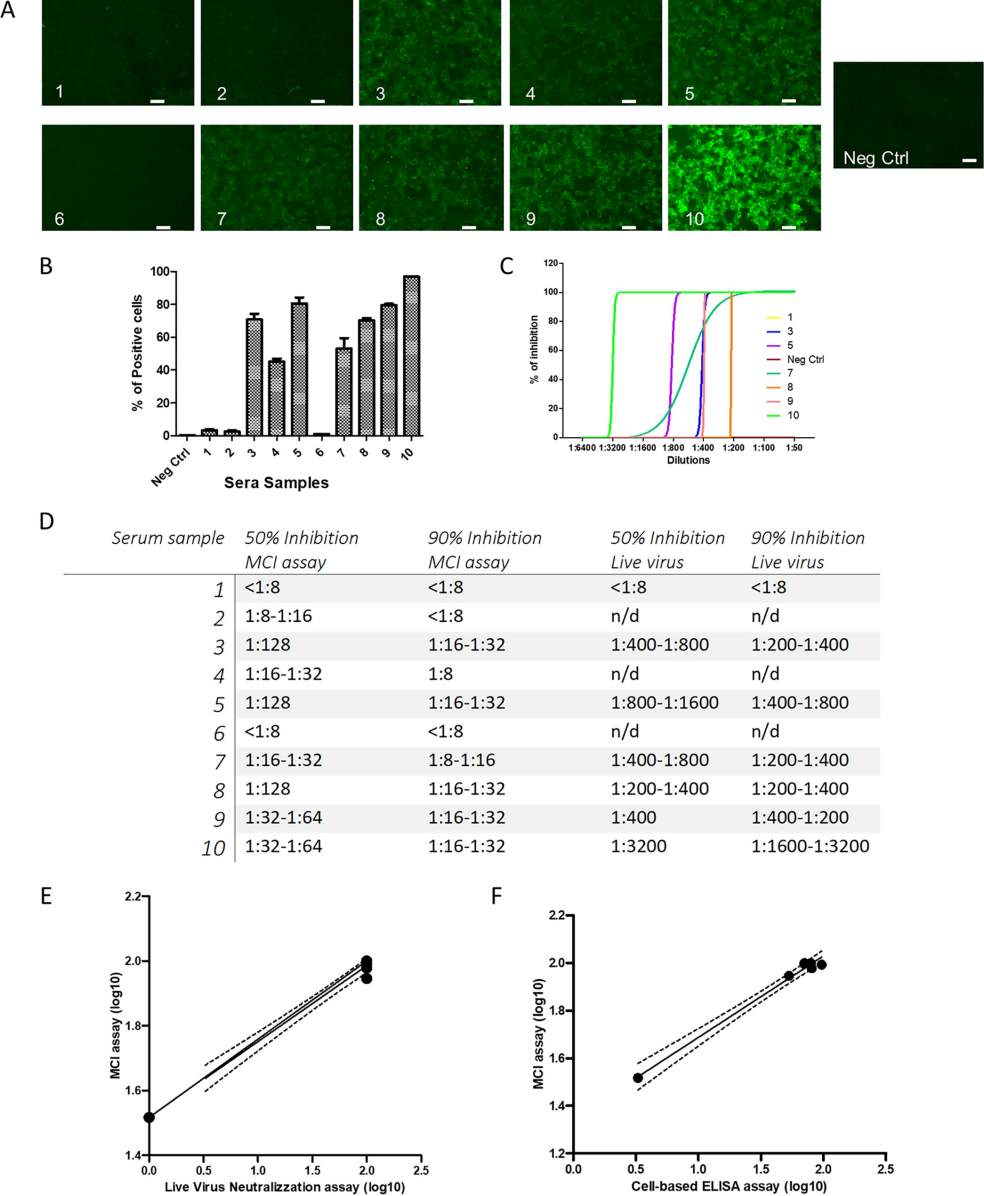
Cell-based immunofluorescent (CBI) and MCI assays to assess SARS-CoV-2 convalescent-phase sera. (A and B) HEK293T cells transiently transfected with the plasmid encoding S were incubated with the indicated sera (diluted 1:20 at 4°C for 1 h, followed by incubation with protein G Alexafluor 488 (1:500) as secondary antibody. Selected fields are shown in panel A. The percentage of positive cells (average from three independent experiments ± SEM) is quantitated in panel B. (C) Selected sera (1, 3, 5, 7–10) were tested for neutralization activity against live SARS-CoV-2 virus. The graph shows the results of a single experiment representative of the three biological replicates. (D) Table comparing the dilutions for 50% and 90% inhibition in MCI and live virus assays for the selected sera. (E) Correlation between the MCI and live virus neutralization data showing serum samples 1, 3, 5, 7, 8, 9, and 10. (F) Comparison of MCI and cell-based ELISA results. Sera with the best binding and neutralization activity (samples 3, 5, and 7 to 10) and a negative control (sample 1) were used. The dotted lines represent the confidence intervals (95% CI).

The cell-based immunofluorescent (CBI) assay provides direct information on clinical serum neutralization activity, requires even less time than the VSV-based pseudotyped virus assay (which requires 48 h for ideal quantitative RFP readings), and is performed in BSL2 conditions. We recently showed that such a CBI assay identifies sera that react to SARS-CoV-2 S (19). We compared ten deidentified sera from SARS-CoV-2 convalescent patients to serum from an uninfected person in CBI, MCI neutralization, and live virus neutralization assays. The ten sera were first assessed using a regular enzyme-linked immunosorbent assay (ELISA) (Fig. S1) then assessed with a CBI assay (Fig. 2A and B). For the experiment in Fig. 2A and B, HEK293T cells expressing the SARS-CoV-2 S protein were incubated with 1:20 dilutions of the indicated sera for 1 h at 4^°^C, after which binding was assessed by fluorescence microscopy (Fig. 2A) and high content image quantitation (Fig. 2B). Selected sera that tested positive in the CBI assay (samples 3, 5, 7, 8, 9, and 10), along with the uninfected patient serum (sample 1) were assessed for live virus microneutralization (Fig. 2C, see also Fig. S2). Figure 2D shows 50% and 90% inhibition in the MCI assay (see also Fig. S3), as well as live virus neutralization activity. Samples 3, 5, and 10 had the most potent neutralization activity. The order of neutralization activity was the same in the MCI and live virus neutralization assays (Fig. 2E). The order of S cell surface recognition in the CBI and MCI assays was also the same (Fig. 2F).

### Live virus neutralization activity correlates with recognition of structural proteins.

**FIG 3:**
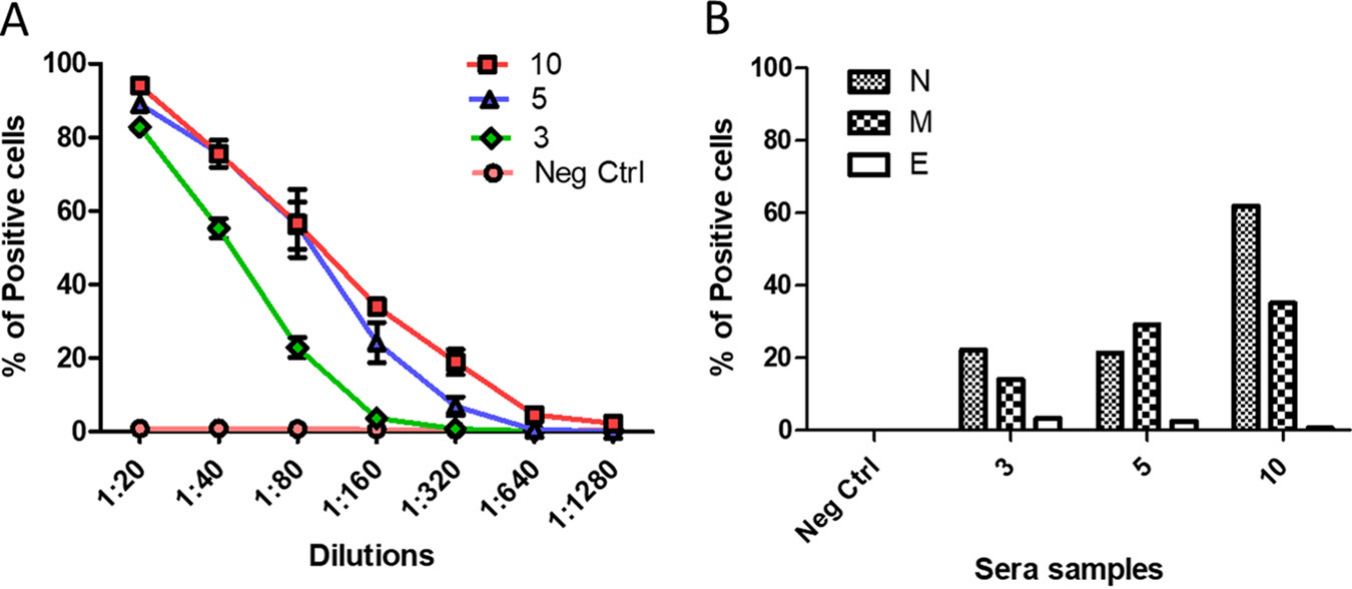
Cell-based immunofluorescent (CBI) assay for the SARS-CoV structural proteins S, E, N, and M. (A) HEK293T cells transiently transfected with plasmid encoding S were incubated with serial dilutions of serum samples 1, 3, 5, or 10 for 1 h at 4°C, followed by incubation with protein G Alexafluor 488 (1:500). Data are shown as the average from three independent experiments ± SEM. (B) HEK293T cells transiently transfected with plasmids encoding viral structural proteins E, N, or M were permeabilized and incubated with serial dilutions of serum samples 1, 3, 5, or 10 for 1 h at 4°C, followed by incubation with protein G Alexafluor 488 (1:500). Data shown are from a representative experiment (from three separate experiments).

We observed that neutralizing activity occurred at higher dilutions in the live virus assay compared to the MCI. The live virus assay relies on a finite number of entry events (plaque-forming units), but the MCI assay mimics several rounds of infection. While these features could account for the observed difference, we considered the possibility that the sera could block viral entry for live virus by interfering with other viral structural proteins, while the MCI assay is based solely on S. Therefore, we assessed whether the neutralization activity correlated with the recognition of S or other SARS-CoV-2 structural proteins. In Fig. 3A, HEK293T cells were transfected with the SARS-CoV-2 S and incubated with serial dilutions of the selected sera. The S recognition persisted at lower dilutions than live virus neutralization (Fig. 2C and D and Fig. 3A). Additionally, the difference between serum samples 5 and 10 was minimal in Fig. 3A, but the neutralization activity of sample 10 against live virus was higher than that of sample 5. This could be attributed to qualitative differences (e.g., increased maturation) between the antibodies present in the two clinical samples.

To search for antibodies against other viral structural proteins in the live virus neutralization activity, we adapted our CBI assay to the other SARS-CoV-2 structural proteins—nucleoprotein (N), matrix protein (M), and envelope protein (E). For the experiment in Fig. 3B, HEK293T cells expressing N, M, or E were permeabilized and incubated with the indicated sera and processed as in Fig. 2A. The best neutralizing serum (sample 10) had significantly more anti-N antibodies than the other two sera (3 and 5) in the CBI assay.

### Validation of the CBI assay using deidentified intensive care unit patient sera.

**FIG 4:**
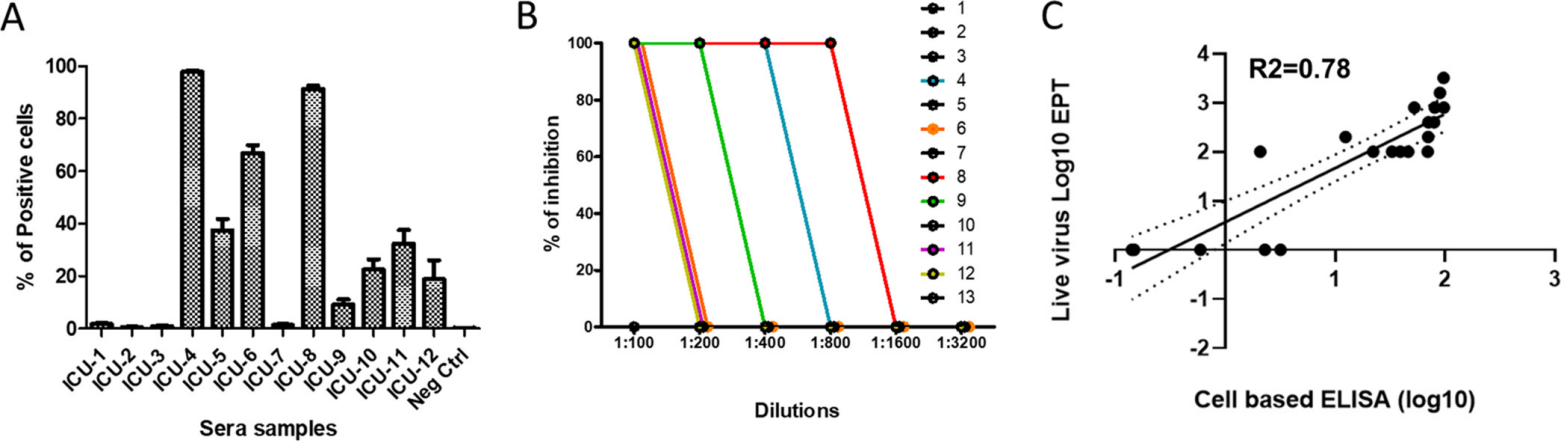
Cell-based immunofluorescent (CBI) assay on SARS-CoV-2 S correlates with antiviral activity with live virus. (A) HEK293T cells transiently transfected with the plasmid encoding S were incubated with sera (diluted 1:20) from 12 ICU patients and 1 negative control at 4°C for 1 h, followed by incubation with protein G Alexafluor 488 (1:500). The % of positive cells (average from three independent experiments ± SEM) is quantitated. (B) The sera from panel A were tested for neutralization activity against live SARS-CoV-2 virus. (C) Regression analysis shows correlation between cell-based immunofluorescent (CBI) assay shown in panel A and a live virus microneutralization assay (*n* = 20). The dotted lines represent the confidence intervals (95% CI).

We have previously provided statistical validation of the correlation between the MCI assay and live virus neutralization (19). To assess the predictive value of the CBI assay in a hospital setting, we used deidentified sera from severely ill patients in the intensive care unit (ICU) (Table 1). A total of 13 samples (12 from ICU patients and 1 from an uninfected person) were assessed in our cell-based assay (Fig. 4A). Additionally, the 12 ICU sera plus the negative control serum were assessed in a live virus microneutralization assay (Fig. 4B). Figure 4C shows the correlation (*R*^2^ = ∼0.78, see Fig. S4) between the live virus microneutralization and the CBI assay results (for S protein) using the 7 samples from Fig. 2C and the 13 samples from Fig. 4B. These 20 samples show agreement between S recognition and the live virus data with a *P* < 0.0001 (Fig. S4).

**TABLE 1:** Infection status of the individuals and the severity of the disease of the positive ones

Serum sample number	Status	Severity of symptoms
1	Positive	−
2	Positive	++
3	Positive	+++/ICU
4	Positive	+++/ICU
5	Positive	+++/ICU
6	Positive	+++/ICU
7	Positive	+++/ICU
8	Positive	+++/Death
9	Positive	+++/ICU
10	Positive	+
11	Positive	+
12	Positive	+
13	Negative	−
14	Negative	−
15	Negative	−
16	Negative	−
17	Negative	−
18	Negative	−
19	Negative	−
20	Negative	−
21	Negative	−
22	Negative	−
23	Negative	−
24	Negative	−

## DISCUSSION

In adapting our multicycle infection (MCI) mimicking assay (8) to SARS-CoV-1 and SARS-CoV-2, we found that the SARS-CoV-2 wild-type (wt) S protein permitted robust quantitation without any modification. For SARS-CoV-1, adding a new cleavage site at position amino acid (aa) 661 to 669 and the mutations P794R and T795R to increase the furin-like protease cleavability was helpful. It is possible that complementation with TMPRSS2 or other host proteases may remove the need for these mutations in the assay for SARS-CoV-1 (or other betacoronaviruses), and we will address this question by adding host proteases in future work.

The MCI assay is simple, robust, and does not require generation of an infectious clone, unlike other recent reports, in which the recombinant VSV virus encoding the SARS-CoV-2 underwent internal evolution to adapt to the VSV background (4). The reported adaptations in the recombinant VSV did not seem to affect the validity of the recombinant virus as a screening tool for neutralizing sera (4); however, recombinant VSV encoding S do not have the flexibility to quickly adapt to newly emerging variants, e.g., D614G (11–13) or recently emerging variants (25, 26). The classic single-cycle infection pseudotyped viral entry assay system requires advance preparation of specific pseudotyped viruses and is therefore not as flexible as the assay described here. The MCI assay can only screen for inhibitors of viral entry and does not interrogate other elements of the replication cycle, since neutralization in this assay is based only on the action of anti-S antibodies (a defect shared with existing pseudotyped viral entry assays).

The CBI assay complements the MCI in that it assesses the humoral response versus all SARS-CoV-2 structural proteins. In either 96- or 384-well (HTS-amenable) plate formats, we envision this assay to be useful for assessing neutralization titers from convalescent plasma donors, to evaluate humoral immunity (from natural infection or from vaccination), and to evaluate antiviral strategies. It can directly assess neutralization by convalescent COVID-19 patient sera as vaccination efforts are under way. It is unclear at present to what degree the vaccination of patients who already have COVID-19 antibodies is of benefit, and in settings of limited vaccine supply or widespread infection, assessing the presence or absence of neutralizing antibodies in such an assay could be a useful public health measure. Assays could be performed pre- and postvaccination in antibody-positive individuals to determine the benefit of vaccination in this group.

In the CBI assay, the most neutralizing serum (sample 10), in addition to binding S, recognized the N protein. In the regular ELISA, patient serum samples 3, 5, and 10 all demonstrated N recognition (Fig. S1), but in the CBI assay, only sample 10 showed N protein binding (Fig. 3B). Future studies will explore the significance of these findings. The CBI assay we present here has several advantages over the classical ELISA in that it does not require production and purification of proteins to coat the plate, and transfection allows for presentation of the full proteins as antigens, including the hydrophobic regions that are often excluded from classical plate-based assays. Proteins expressed on the surface of live cells offer the most native conformation of the antigens for assessment of sera. Any change due to viral evolution can be quickly assessed. The suitability of this assay for BSL2 conditions means that upscaling and widespread use for clinical decision making could be straightforward (27–35). In light of the current vaccines that are aimed at raising an anti-S protein immune response, and recent evolution in the S gene leading to new mainstream viral variants, we propose our strategy as a useful screening method. The finding that the CBI assay results directly correlate with live virus neutralization activity suggests that this assay could serve for a first-pass assessment of neutralization activity of sera under BSL2 conditions.

## MATERIALS AND METHODS

### Cells and virus.

Cell lines HEK293T (human kidney epithelial, ATCC CRL-3216) and Vero (African green monkey kidney, ATCC CCL-81) were purchased from ATCC. Cells were grown in Dulbecco’s modified Eagle’s medium (DMEM + GlutaMAX, Gibco) supplemented with 10% fetal bovine serum (HI FBS, Gibco,) and 1% penicillin streptomycin (Pen Strep, Gibco) antibiotics at 37°C in 5% CO_2_. VSV-ΔG-RFP pseudotyped with VSV-G was derived from the cDNA of VSV Indiana, in which the surface glycoprotein G gene has been replaced with the reporter RFP gene.

### Plasmids and peptides.

The genes encoding SARS-CoV-1 S and SARS-CoV-2 S, M, N, E, VSV-G, and hACE-2 proteins were custom synthesized by Epoch Biosciences, Inc. and cloned into a pCAGGS-puromycin vector. For SARS-CoV-1 S_New Cleav P794R T795R_, a new cleavage site for furin (position 661 to 669) and two mutations (P794R and T795R) were introduced into the SARS Urbani S_wt_ sequence. For SARS-CoV-1 S_P794R T795R_, only the reported mutations were introduced. SARS and MERS lipopeptides have been described (23).

### Human sera.

Serum samples were obtained from quantitative reverse transcriptase PCR (RT-qPCR)-positive patients with COVID-19 symptoms ranging from mild to severe (Table 1). Sera were collected by routine phlebotomy and the samples were heat-inactivated at 56°C for 30 min before use. Information about the 20 samples from an Italian hospital is presented in Table 1.

### Cell-based immunofluorescent (CBI) assay.

Aliquots of 4 × 10^4^ 293T cells were seeded in 96-well plates the day before transfection. Cells were transfected with the plasmid encoding the indicated coronavirus structural proteins or empty vector (negative control). For the cell-based assay experiments to assess S binding, 24 h after transfection the cells were incubated with the sera for 1 h at 4°C to allow antibody binding. For the ELISA experiments to assess binding to E, M and N proteins, cells were permeabilized with 0.1% Triton X-100 before incubation with sera. HEK293T cells were washed three times with Dulbecco’s phosphate-buffered saline (DPBS) and fixed with 4% paraformaldehyde (PFA). Protein G Alexafluor 488 was used as a secondary antibody. The plates were imaged using an InCell Analyzer 2000 instrument (GE Healthcare). Images were acquired using the DAPI (nuclei, 4′,6-diamidino-2-phenylindole) and fluorescein isothiocyanate (FITC) channels (Alexa 488 signal); four fields per well covering the whole well were imaged using the 4× objective. The fluorescence signal was quantified using Cell profiler and Knime software. Serial dilutions of sera were used to evaluate the sensitivity of the assay and to assess the accuracy of the percentage of positive cell determination.

### Enzyme-linked immunosorbent assay (ELISA).

High protein absorbance 96-well plates (Nunc) were coated with recombinant (nonglycosylated RBD, S1, and N antigens (all from Ray Biotech, Inc.) and glycosylated S_1_ and S_2_ complex (kind gift of Filippo Mancia) in carbonate buffer at 100 ng/50 μl/well overnight at 4°C. After washing with PBS, the plates were blocked with 0.5% Carnation milk in PBS, 300 μl/well at room temperature (RT) for 1 h. Patients’ sera in serial dilutions with blocking solution was incubated with the antigens at 50 μl/well at RT for 1 h. After washing with PBS, HRP-conjugated goat anti-human IgG was added to the well and incubated at RT for 1h. After thorough washing, 50 μl/well horseradish peroxidase (HRP) chromogenic substrate 3,3′,3’5-TMB (Sigma) was added. The reaction was stopped after 20 to 25 min of incubation with 20 μl of 1N HCl and absorbance (A) values were measured at 452 nm using MULTISCAN MCC microplate reader (Thermo Fisher Scientific). Values of 2-fold of the mean absorbance from blank wells containing blocking solution were used as a cutoff for the endpoint of antibody titer calculation. All experiments were performed in duplicates.

The data are presented in reference to normal sera from 3 individuals who never had SARS-CoV-2 infection. Binding to SARS-CoV-2 antigens was normalized against average background binding of normal sera at serum dilutions of 1:400. This dilution point was chosen since it falls on a linear part of the titration curve generated in an ELISA. Data represent mean values or mean values with standard deviation (SD). Significant differences between means of individual serum samples were tested using a one-way analysis of variance (ANOVA).

### Pseudovirus neutralization assay.

We previously developed a pseudovirus-based neutralization assay to assess inhibition of infection by high biocontainment enveloped viruses under low-level biocontainment (8) and we adapted it for SARS CoV. SARS-CoV-1 and -2 S proteins were pseudotyped onto recombinant vesicular stomatitis virus (VSV) that expresses red fluorescent protein (RFP) but does not express the VSV attachment protein G (VSV-ΔG-RFP). HEK293T (human kidney epithelial) cells were cotransfected with full-length codon-optimized SARS-CoV-1 or -2 S protein (Epoch Life Science, Missouri City, TX), the viral entry receptor hACE2 (Epoch Life Science, Missouri City, TX), and green fluorescent protein (GFP). These transfected HEK293T cells were then infected with VSV-ΔG-RFP pseudotyped with VSV-G at a multiplicity of infection (MOI) of 0.02 for 1 h. The cells were mixed at a 2 to 1 ratio with Vero (African green monkey kidney) cells, which have high endogenous expression of hACE2 (36). Vero cells were overlain on top after infection to increase the signal; without this step, RFP values were significantly lower. The cells were then combined with dilutions of serum or plasma in 96-well plates. The infected S protein-expressing HEK293T cells generate VSV-ΔG-RFP viruses that bear S protein. These viruses infect and drive RFP expression in Vero cells, and undergo multiple cycles of entry and budding in the HEK293T cells due to the coexpression of S protein with hACE2. The GFP and RFP signals were measured at the indicated time points after plating (Infinite M1000 PRO microplate reader, Tecan, Männedorf, Switzerland), with robust amplification of the S protein pseudovirus-driven RFP signal, derived from both the cells producing the pseudotype and the ACE-2-expressing cells, at 24 to 48 h. Loss of RFP signal amplification indicates S protein neutralizing activity in patient plasma. Identical, 2-fold serial dilutions were performed for all samples and there were no missing titration data points for any of the samples.

### SARS-CoV-2 viral stock production.

SARS-CoV-2 (2019-nCoV/USA_WA1/2020) was provided to B.H. by the World Reference Center for Emerging Viruses and Arboviruses (WRCEVA) (Galveston, TX, USA). To generate virus stocks, Vero E6 cells (provided by F.-L. Cosset) were inoculated with virus at an MOI of 0.01. Supernatant fluid was harvested at 72 h postinfection, clarified by low-speed centrifugation, filtered at 0.45 μm, aliquoted, and stored at −80°C. Virus stock was quantified by limiting dilution plaque assay on Vero E6 cells, as described (19).

### Live virus neutralization assay.

Two-fold dilutions of plasma in 50 μl of Dulbecco’s modified Eagle medium (DMEM) were incubated with 200 plaque-forming units (PFU) of SARS-CoV-2 in 50 μl of DMEM for 30 min at 4°C. Aliquots of 100 μl of DMEM + 4% FBS containing 3 × 10^4^ Vero E6 cells were added to achieve a final dilution of sera from 1:50 to 1:6,400 (4 wells per dilution). Cells were incubated for 5 days at 37°C, 5% CO_2_. Cytopathic effect was revealed by crystal violet staining and scored by an observer blinded to the study design and sample identity. Neutralization endpoint titers were expressed as the value of the last serum dilution that completely inhibited virus-induced cytopathic effect.

### Statistical analyses.

Data are shown as mean ± standard error of the mean (SEM) of three independent experiments. Simple linear regression analysis was performed to assess correlation between the live virus microneutralization and cell-based immunofluorescent (CBI) assays using log_10_ of the microneutralization endpoint titer (last dilution at which 25% or greater of wells showed complete inhibition of cytopathic effect) and log_10_ of the percentage of S protein-expressing cells that stained positive for human IgG. Student’s *t* test was applied to calculate the statistical significance. To evaluate the correlation between MCI and live virus seroneutralization data, we performed a linear regression analysis. Log_10_ of the percentage of inhibition of infection was used for both assays. The correlation between the MCI and the CBI assay results was assessed in a similar manner using log_10_ of the percentage of inhibition of infection and log_10_ of the percentage of S protein-expressing cells that stained positive for human IgG, respectively. Correlation of the CBI assay with the classical ELISA approach was analyzed through a simple linear regression analysis. Statistical analysis was performed using Prism software (v8, GraphPad).

### Ethical statement.

Serum samples were collected in accordance with Columbia University IRB-approved protocol (AAAT0368). ICU serum samples were collected in Italy according to the IRB-approved protocol (140/20/ESCOVID19).

10.1128/mSphere.00571-21.1 TABLE S1 IC_50_ and IC_90_ values of SARS-CoV-2-peg4-Chol and MERS-peg4-Chol peptides on VSV-RFPΔG*/SARSCoV-2 S pseudotyped virus and SARS-CoV-2 virus. Download Table S1, PDF file, 0.03 MB. Copyright © 2021 Stelitano et al. 2021 Stelitano et al. https://creativecommons.org/licenses/by/4.0/ This content is distributed under the terms of the Creative Commons Attribution 4.0 International license.

10.1128/mSphere.00571-21.2 FIG S1 Serum antibody titers assessed by ELISA. (A) Donor numbers 1, 2, and 6 at the time of blood collection were COVID-19 negative as per RT-qPCR and had no symptoms. The other individuals were recovered; negative at the time of blood collection, but positive and symptomatic at one to two months earlier. For each indicated antigen, the ratio between the sample serum at 1:400 dilution and the negative control serum (at the same dilution) is presented in the *y* axis (optical density at 452 nm [OD_452_] of the sample/OD_452_ of a mean normal control). Error bars are given based on duplicate measurements. The normal control is an average of three normal sera collected from uninfected individuals. (B) Correlation between the data in A and the data from the CBI assay in Fig. 2B. Points are from the sera with the best binding (sera 3, 5, 7, 8, 9, and 10) and a negative control (serum 1). The dotted lines represent the confidence intervals (95%). Download FIG S1, TIF file, 0.3 MB. Copyright © 2021 Stelitano et al. 2021 Stelitano et al. https://creativecommons.org/licenses/by/4.0/ This content is distributed under the terms of the Creative Commons Attribution 4.0 International license.

10.1128/mSphere.00571-21.3 FIG S2 Neutralization activity of human sera with live virus in BSL3. The sera from SARS-CoV-2-infected or exposed individuals were screened for neutralization activity using live virus. Dose response curves with serial dilutions (*x* axis). Data points are the same as those presented in Fig. 2C. Download FIG S2, TIF file, 0.2 MB. Copyright © 2021 Stelitano et al. 2021 Stelitano et al. https://creativecommons.org/licenses/by/4.0/ This content is distributed under the terms of the Creative Commons Attribution 4.0 International license.

10.1128/mSphere.00571-21.4 FIG S3 Neutralization activity of human sera in MCI. The sera from SARS-CoV-2-infected or exposed individuals were screened for neutralization activity using the MCI assay. Dose response curves with serial dilutions (*x* axis). Download FIG S3, TIF file, 0.2 MB. Copyright © 2021 Stelitano et al. 2021 Stelitano et al. https://creativecommons.org/licenses/by/4.0/ This content is distributed under the terms of the Creative Commons Attribution 4.0 International license.

10.1128/mSphere.00571-21.5 FIG S4 Statistical analysis of plasma neutralizing activity. The endpoint titers in a live virus microneutralization assay were correlated with the cell-based assay results (*n* = 20) using linear regression analysis. Data are from Fig. 4C. The dotted lines represent the confidence intervals (95%). Download FIG S4, TIF file, 0.3 MB. Copyright © 2021 Stelitano et al. 2021 Stelitano et al. https://creativecommons.org/licenses/by/4.0/ This content is distributed under the terms of the Creative Commons Attribution 4.0 International license.
