# Supplementary material for: Rapid and Flexible Platform To Assess Anti-SARS-CoV-2 Antibody Neutralization and Spike Protein-Specific Antivirals
Source: mSphere. 2021 Jul 28;6(4):e00571-21. doi: 10.1128/mSphere.00571-21 (PMC8386372; doi:10.1128/mSphere.00571-21)
Supplement: TABLE S1 [file msphere.00571-21-st001.pdf]

Peptide	***IC_{50} (nM)***	***IC_{90} (nM)***	***IC_{50} (nM) virus***	***IC_{90} (nM) virus***
SARSCoV-2-peg4-Chol	0.0423±0.03	3.8±0.10	8.9±2.1	102±25
MERS-peg4-Chol	0.61±0.15	25.5±12.33	115±33	1000±72

Table S1. Table showing the IC_{50} and IC_{90} of SARS-CoV-2-peg4-Chol and MERS-peg4-Chol peptides on VSV-RFPΔG*/SARSCoV-2 S pseudotyped virus and SARS-CoV-2 virus.
