# Supplementary figures and images for: Rapid and Flexible Platform To Assess Anti-SARS-CoV-2 Antibody Neutralization and Spike Protein-Specific Antivirals
Source: mSphere. 2021 Jul 28;6(4):e00571-21. doi: 10.1128/mSphere.00571-21 (PMC8386372; doi:10.1128/mSphere.00571-21)

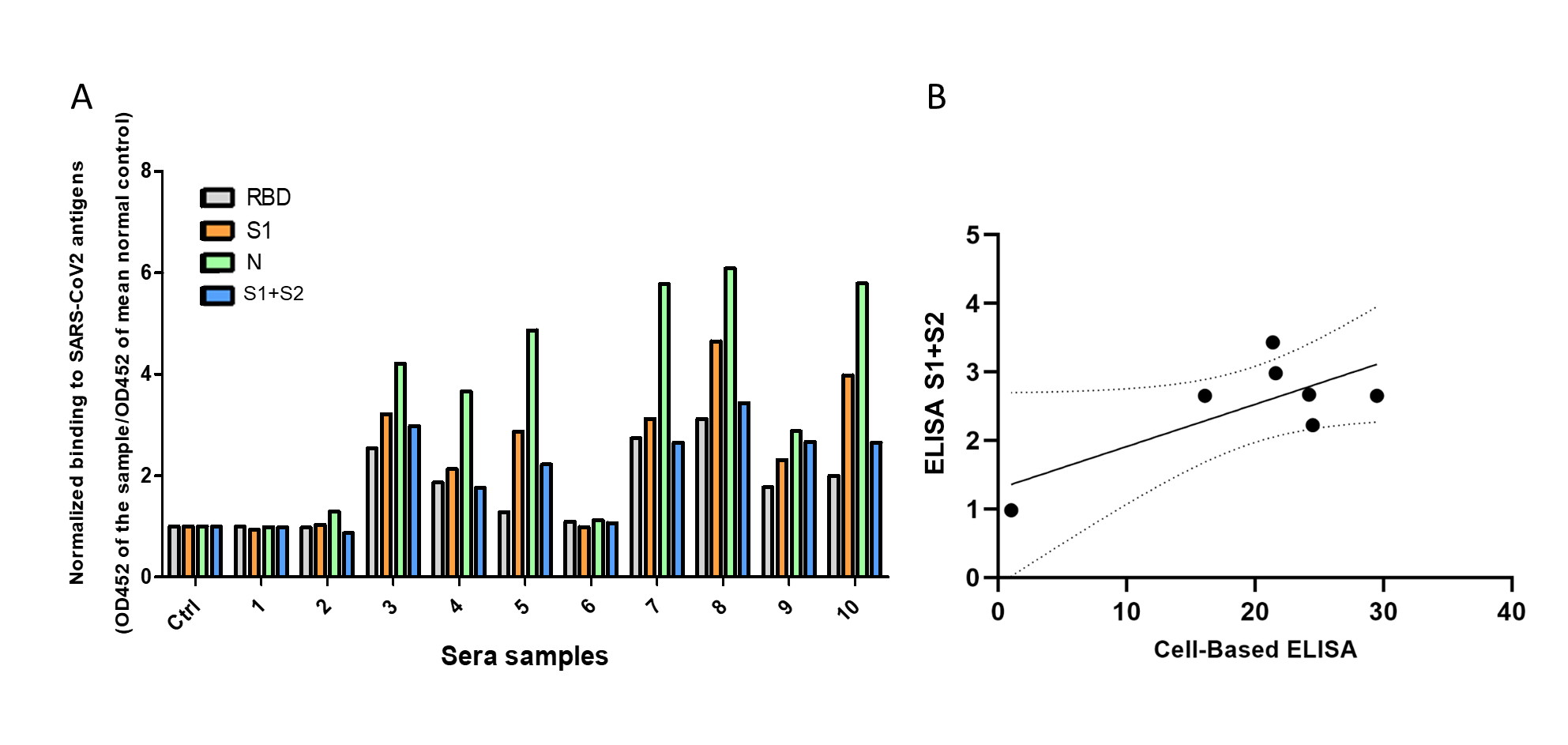

Supplement: FIG S1 [file msphere.00571-21-sf001.tif]

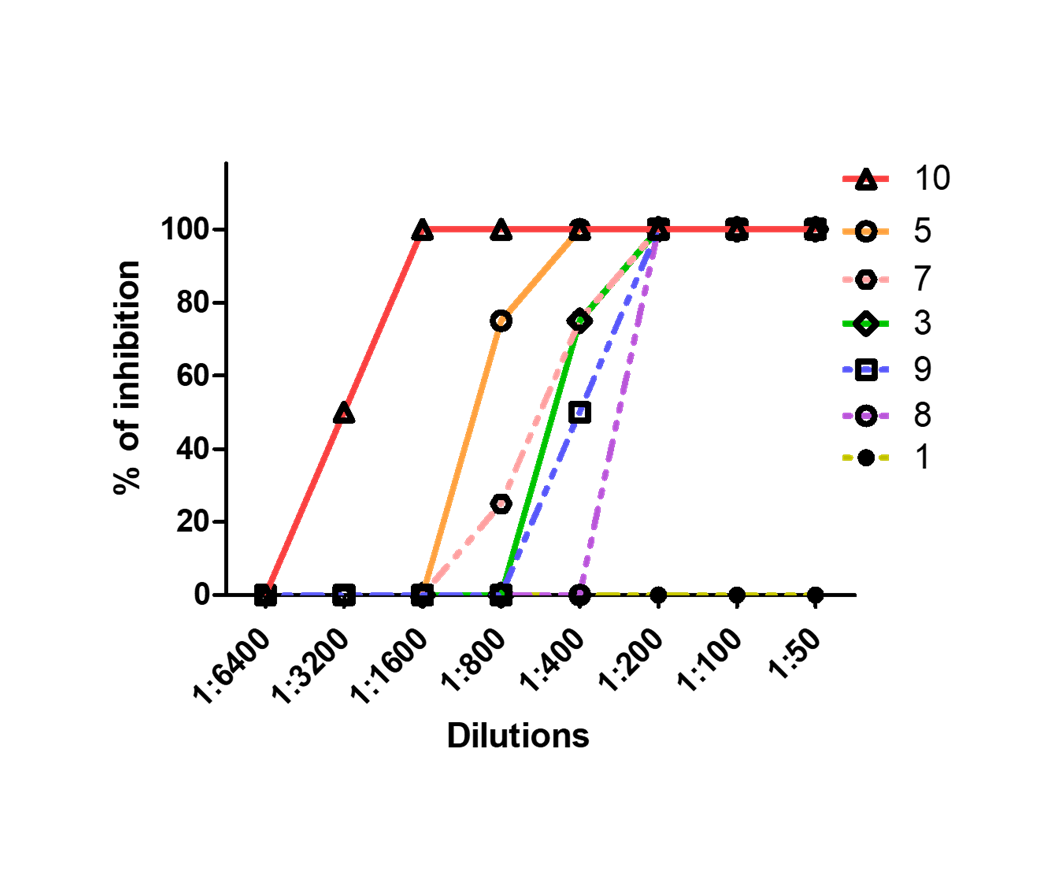

Supplement: FIG S2 [file msphere.00571-21-sf002.tif]

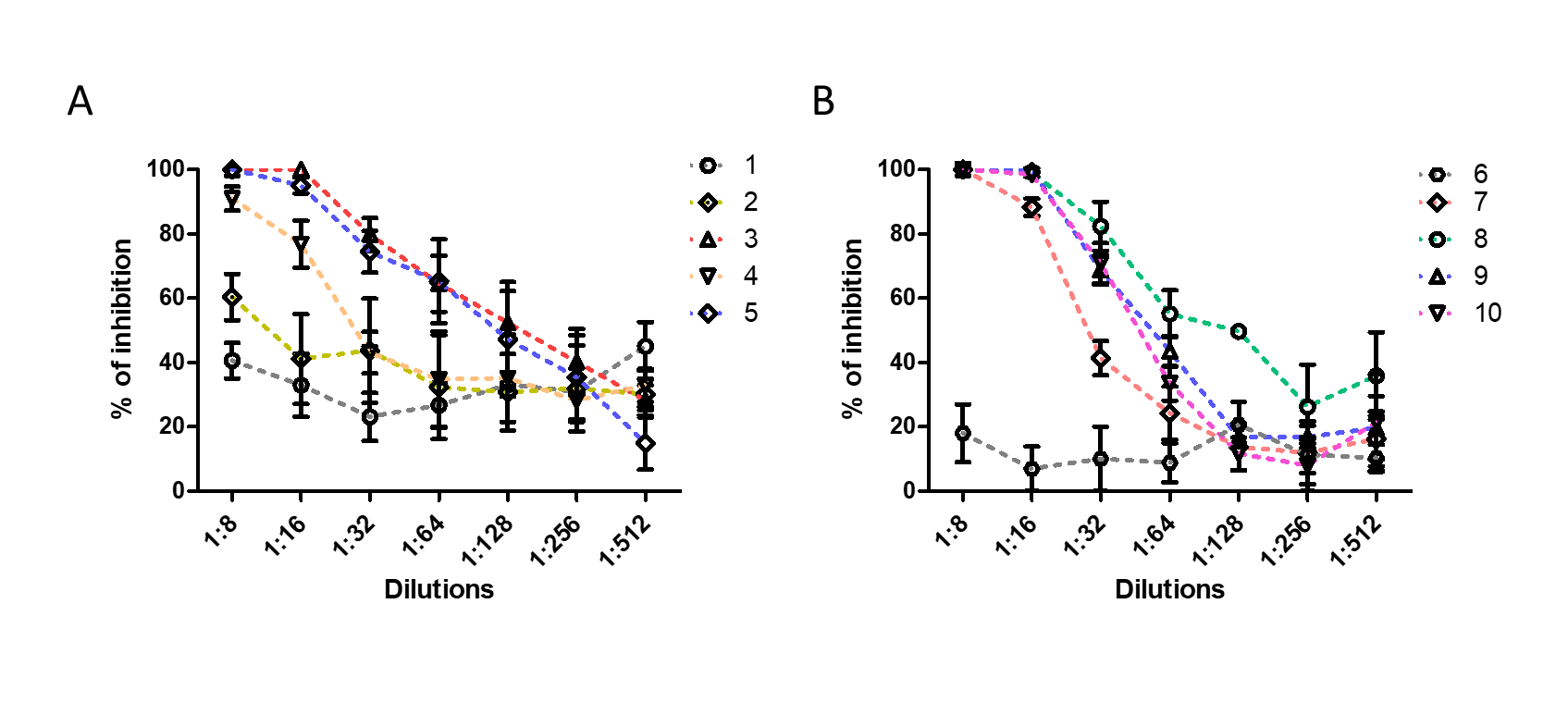

Supplement: FIG S3 [file msphere.00571-21-sf003.tif]

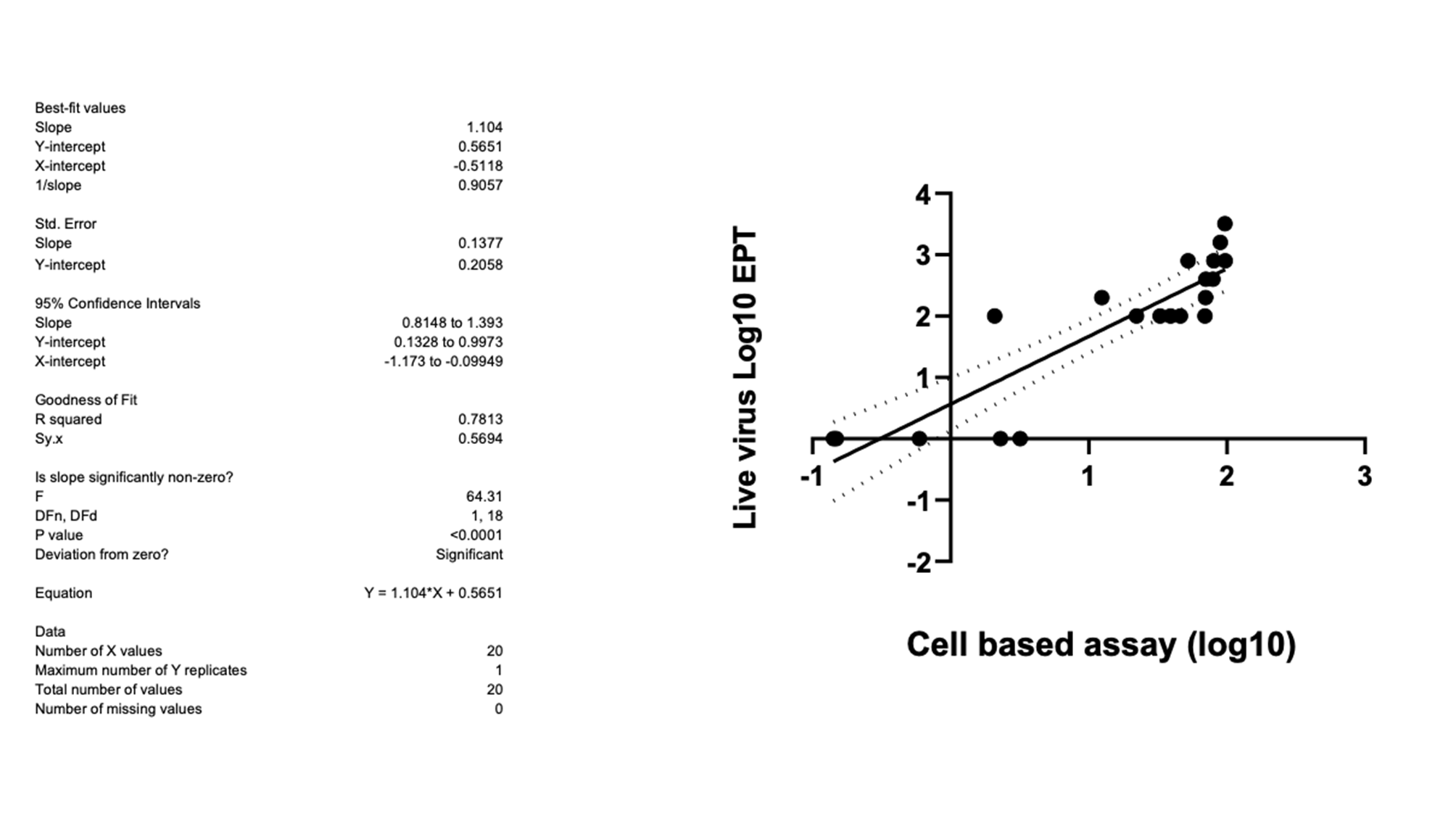

Supplement: FIG S4 [file msphere.00571-21-sf004.tif]
